# Supplementary material for: Multigene phylogeny and morphological descriptions of five species of Agaricus sect. Minores from subtropical climate zones of Pakistan
Source: PLoS One. 2024 Jul 11;19(7):e0302222. doi: 10.1371/journal.pone.0302222 (PMC11239060; doi:10.1371/journal.pone.0302222)
Supplement: Attachment — Submitted filename: PONE review 2024.docx [file pone.0302222.s001.docx]

Title of the paper: Multigene phylogeny and morphological descriptions of five species of Agaricus sect. Minores from subtropical climate zones of Pakistan

**Abstract**:

First two sentence of the abstract seems to be incomplete. Rewrite it.

Use the full form of ‘nuc’ in nuc rDNA.

What about the morphological description of the mentioned five species, you have only mentioned the geographical description. Kindly provide in brief about the molecular and morphological description of the mention five species.

At the end of the abstract put a sentences about the importance of the study and it will be useful.

**Introduction**: In Introduction part, although the authors has tried to explain in details about the mushroom species, the sentences are unclear. The aim/objectives of the study has not been mentioned.

Last sentence of the introduction should be in materials and method in subheading specimen collection and sampling sites

**Materials and methods:**

Subheading ‘Specimen collection ….’ The country name can be removed as it’s not required in the subheading.

Kindly explain the method of collected of the specimens and preservation/ fixation method for molecular analysis.

2^nd^ sentence ‘based on morphological observations………’ should be in result part.

Kindly put coordinates for the locations and details of each location can be placed in proper way in the form of a table.

Subheading: Morphological observations- Correct the word ‘characeterization’

Mention the magnification of microscope used for measurement

Kindly provide the details of sequence submitted in MycoBank as a supplementary table.

Subheading ‘DNA sequence alignments and phylogenetic analyses’: correct the word ‘phylogeentic’ Mention the data analysis part in separate subheading

**Results:**

In table 1: Kindly mention the table heading

What does different color indicates in the table, kindly mention in notes.

‘The multigene sequence dataset…….. but still it remains……..data’: sentence in not clear. Modify it.

Why to put word notes there in result subheading. Give a proper subheading for the content.

Discussion:

‘Agaricus cf. goossensiae (ADK2751) was initially used in the phylogenetic analysis by Zhao et al. 2011 [29] later as A. sp.’-complete the sentence, its unclear.

Discussion part needs to be modified, citing more and recent references and comparing with the study.

No conclusion part. Kindly add the conclusion after the discussion

Figure of phylogeny is not at all clear. Replace it with high resolution figure.

Figures of the mushroom species have not been marked properly. Kindly recheck it.
